# 

**Published:** 1842-10-01

**Authors:** 


### MEDICAL EXAMINER,

EDITED BY

*J. B. BIDDLE, M.D. & W. W. GERHARD, M. D.*

VOL. I.] OCTOBER 1, 1842. [No. 40.

